# Cloning and Characterisation of the Gene Encoding 3-Hydroxy-3-Methylglutaryl-CoA Synthase in *Tripterygium wilfordii*

**DOI:** 10.3390/molecules191219696

**Published:** 2014-11-27

**Authors:** Yu-Jia Liu, Yu-Jun Zhao, Meng Zhang, Ping Su, Xiu-Juan Wang, Xia-Nan Zhang, Wei Gao, Lu-Qi Huang

**Affiliations:** 1 School of Traditional Chinese Medicine, Capital Medical University, Beijing 100069, China; E-Mails: lyjlife@sina.com (Y.-J.L.); diana1989119@sina.com (Y.-J.Z.); zhangmeng8846@163.com (M.Z.); suping120@163.com (P.S.); wxj0517@sina.com (X.-J.W.); xnzhang@ccmu.edu.cn (X.-N.Z.); 2 National Resource Center for Chinese Materia Medica, China Academy of Chinese Medical Sciences, Beijing 100700, China

**Keywords:** HMGS, mevalonate (MVA) pathway, *Tripterygium wilfordii*, terpenoids

## Abstract

*Tripterygium wilfordii* is a traditional Chinese medical plant used to treat rheumatoid arthritis and cancer. The main bioactive compounds of the plant are diterpenoids and triterpenoids. 3-Hydroxy-3-methylglutaryl-CoA synthase (HMGS) catalyses the reaction of acetoacetyl-CoA to 3-hydroxy-3-methylglutaryl-CoA, which is the first committed enzyme in the mevalonate (MVA) pathway. The sequence information of HMGS in *Tripterygium wilfordii* is a basic resource necessary for studying the terpenoids in the plant. In this paper, full-length cDNA encoding HMGS was isolated from *Tripterygium wilfordii* (abbreviated *TwHMGS*, GenBank accession number: KM978213). The full length of *TwHMGS* is 1814 bp, and the gene encodes a protein with 465 amino acids. Sequence comparison revealed that *TwHMGS* exhibits high similarity to *HMGS*s of other plants. The tissue expression patterns revealed that the expression level of *TwHMGS* is highest in the stems and lowest in the roots. Induced expression of *TwHMGS* can be induced by MeJA, and the expression level is highest 4 h after induction. The functional complement assays in the *YML126C* knockout yeast demonstrated that *TwHMGS* participates in yeast terpenoid biosynthesis.

## 1. Introduction

*Tripterygium wilfordii* is a widely used traditional Chinese medicine that exhibits anti-inflammatory [1] and anti-rheumatoid arthritis [2] activity. Furthermore, bioactive terpenoids from *Tripterygium wilfordii* were proposed to inhibit growth and induce apoptosis in human cancer cell lines, and have cytotoxic activity [3,4]. The main bioactive chemical compounds of *Tripterygium wilfordii* belong to different families [5]: triptolide and triptophenolide are diterpenes; celastrol and wilforlide A belong to the triterpenoids. Studies showed that these terpenoids affect cell proliferation in tumours by different mechanisms [6,7] and may provide novel means to treat cancer.

**Scheme 1:**
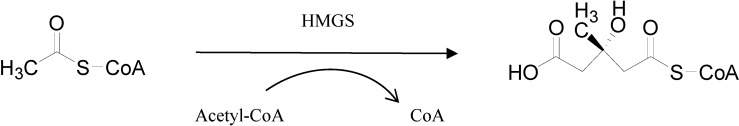
The HMGS-catalyzed chemical reaction.

Terpenoids exhibit a broad range of bioactivities and diverse chemical structures. Tanshinones have been extensively used for the treatment of coronary heart disease, cardiovascular disorders, chronic renal failure and human tumours [8], ginkgolides are widely used for the prevention and treatment of thrombi [9], and glycyrrhizic acid exhibits anti-inflammatory, antitumour, antiulcer, antiviral, antiallergic, anti-dotal, and anti-oxidant biological activity [10]. Two terpenoid biosynthetic pathways are commonly recognised in higher plants: the cytosolic mevalonic acid (MVA) pathway [11], and the plastidic 2-methyl-d-erythritol-4-phosphate (MEP) pathway [12]. There exists cross-talk between the two pathways [13]. However, the contribution of each pathway in the biosynthesis of different terpenoids is variable [14]. The C_5_ units from the MEP pathway feed largely into the formation of C_10_, C_20_ and C_40_ compounds, while the C_5_ units from MVA pathway feed largely into the formation of C_15_, C_25_, C_30_ compounds [15]. HMGS is one of the upstream genes in the MVA pathway that catalyses the formation of the basic C_5_ building block. HMGS is involved in the catalysis of acetoacetyl-CoA to 3-hydroxy-3-methylglutaryl-CoA, which is the first committed step in the MVA pathway (Scheme 1). The analysis of *HMGS* in *Tripterygium wilfordii* is thus important to investigate the biosynthesis of terpenoids active compounds.

The mechanism of action of HMGS has been widely researched by detecting the structures of its reaction intermediates [16,17]. Through the determination of the crystal structure of HMGS bound to different reaction intermediates, the catalytic mechanism of the reaction was determined. This revealed that the carbon-carbon bond formation is facilitated through the activation of the methyl group of an acetylated cysteine [18].

However, the enzymes involved in the biosynthesis of terpenoids in *Tripterygium wilfordii* are not well understood. Because the transcription of HMGS has been proven to be relevant to the accumulation of terpenoids in organisms [19,20], the identification of these enzymes and their genetic sequences are important for further studies of terpenoid biosynthesis in *Tripterygium wilfordii*. Besides, the sequences of the MVA pathway genes in *Tripterygium wilfordii* are necessary to produce bioactive compounds in yeast by synthetic biology strategies. To date, no study has described the genes in the MVA pathway of *Tripterygium wilfordii*. Therefore, we cloned the *HMGS* gene in *Tripterygium wilfordii* and characterised the gene using yeast complement assays. In addition, the tissues expression of HMGS in *Tripterygium wilfordii* aseptic seedlings and the expression in *Tripterygium wilfordii* suspension cells induced by Methyl jasmonate (MeJA) were investigated.

## 2. Results and Discussion

### 2.1. Cloning and Sequence Analysis of TwHMGS

**Figure 1:**
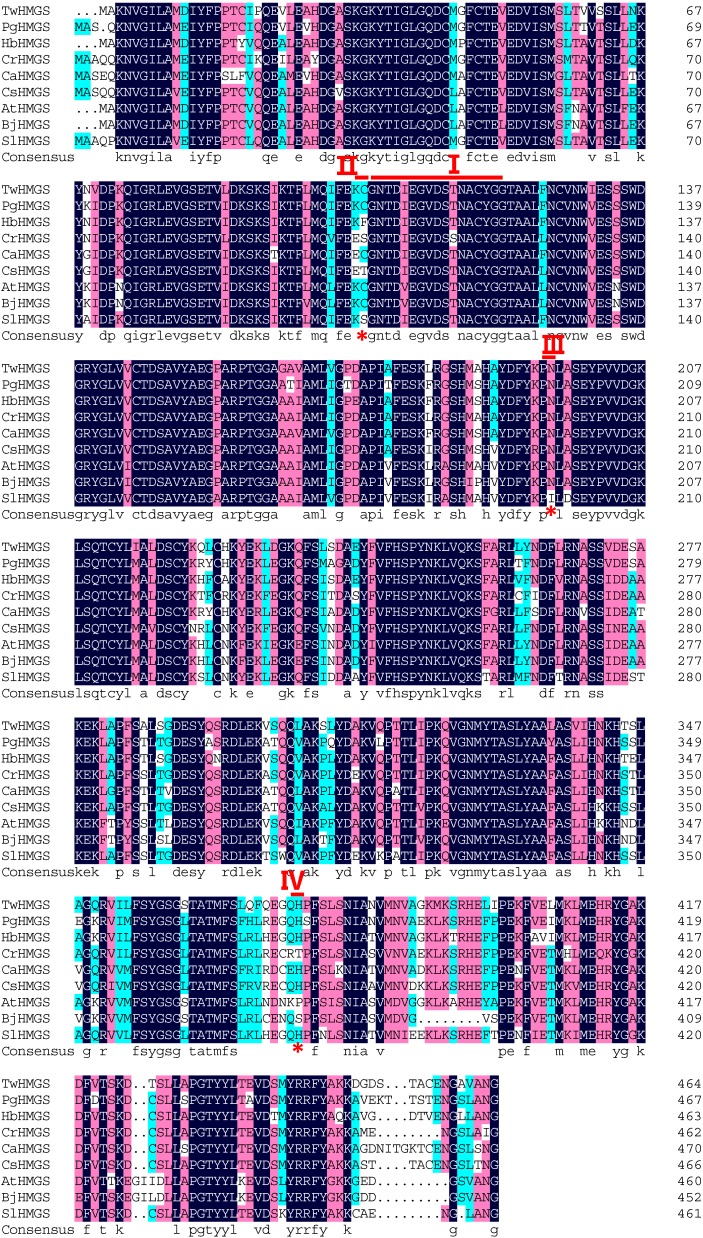
Alignment of *Tw*HMGS with other HMGS genes in plants.

Full length *TwHMGS* is 1814 bp and contains a 1398-bp ORF. The gene encodes a 465-amino acid protein. The multiple alignment analysis demonstrated that *TwHMGS* exhibited high similarity to *HMGS* genes from other plants, including *Panax ginseng* (ADI80347.1, 86%), *Hevea brasiliensis* (BAF98279.1, 86%), and *Brassica juncea* (AAG32924, 80%) (Figure 1). Based on the functional domain analysis, the active site of HMG-CoA synthase active site exists between amino acids 105 and 120 [18].

**Figure 2:**
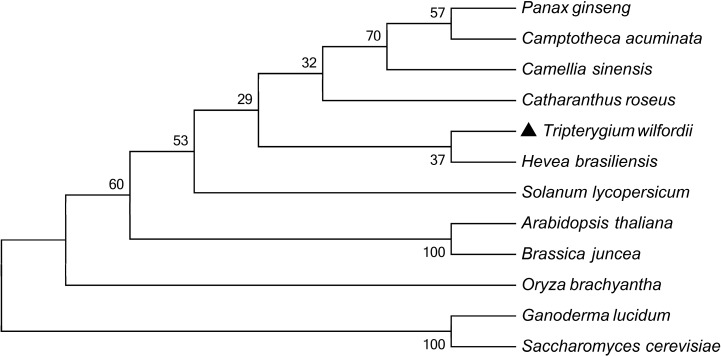
Phylogenetic tree of HMGS from different species using the neighbour-joining method.

**Figure 3:**
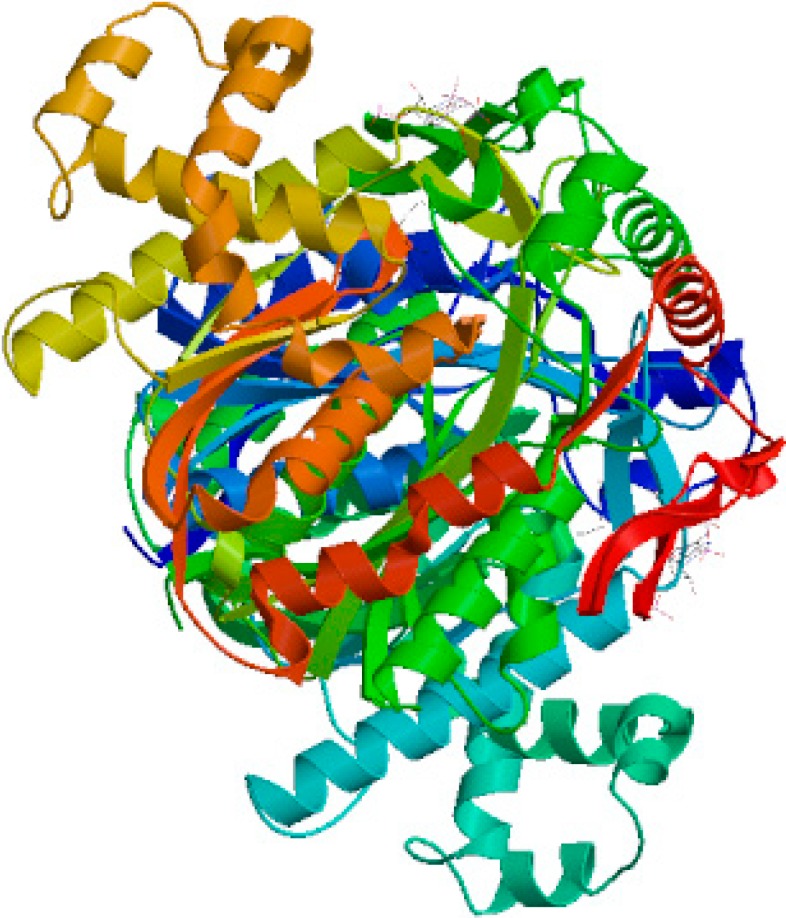
The 3D structure of *Tw*HMGS using homology-based modelling.

The phylogenetic tree was constructed according to the deduced amino acid sequence of *TwHMGS* and other HMGS genes from different hosts (Figure 2). The tree revealed that *TwHMGS* exhibited the highest homology with HMGS from *Hevea brasiliensis*. All of the HMGS genes selected from the plants clustered together, and the HMGS gene from eumycophyta clustered as a different group. 3D modelling by the Swiss-Model (template: *Brassica juncea*, Seq Identity: 83.93%) indicated that the *Tw*HMGS catalytic domain was able to form a homodimer with two acetyl-CoA ligands [21] (Figure 3).

### 2.2. Functional Identification in Yeast

The MVA pathway is essential for yeast survival, and the absence of HMGS in yeast is lethal. YSC6274 is a HMGS-disrupted heterozygotic diploid strain that can grow on YPD + G418 medium.

**Figure 4:**
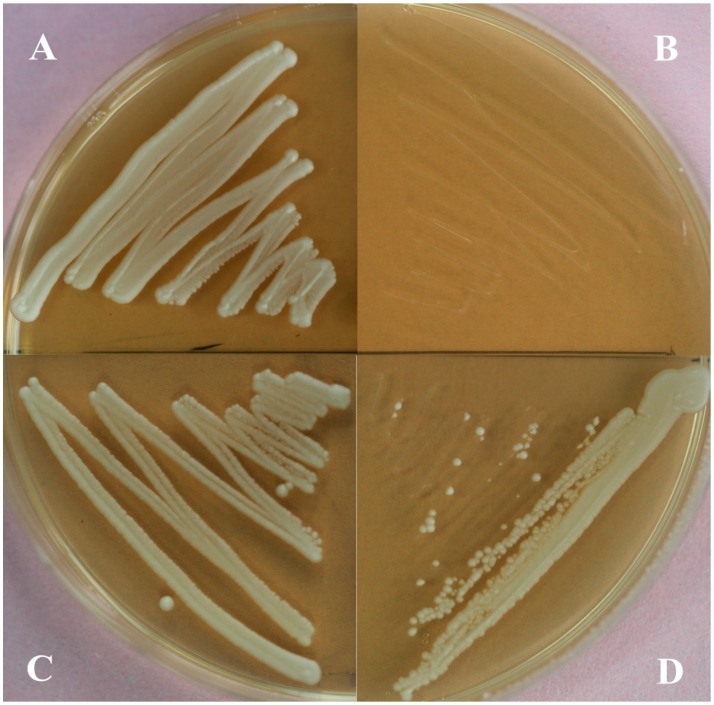
Functional complementation for the growth of the yeast strain YSC6274. (**A**) Diploid YSC6274 on YPD + G418 medium grew within 2 days; (**B**) Haploid YSC6274 containing *pYES2-TwHMGS* on YPD + G418 medium failed to grow; (**C**) Diploid YSC6274 on YPG + G418 medium grew within 2 days; (**D**) Haploid YSC6274 containing *pYES2-TwHMGS* on YPG + G418 medium grew within 3 days.

The haploid YSC6274 transfected with empty pYES2 vector was unable to grow on either YPD or YPG medium. The haploid strains transformed with plasmids were unable to grow on SC (-Ura) + 5-FOA medium. The diploid YSC6274 was able to grow on both YPD + G418 and YPG + G418 medium (Figure 4A,C). However, the haploid HMGS-disrupted strain transfected with the *pYES2-TwHMGS* vector died on YPD+G418 medium (Figure 4B) but was able to survive on YPG + G418 medium (Figure 4D) . These data demonstrate that *Tw*HMGS possesses HMGS activity.

I. The active site of HMGS (105-120); II. *Cys* amino acid residue; III. *Asn* amino acid residue; IV. *His* amino acid residue. * The conserved amino residues. The accession numbers are as follows: *Tw*HMGS (*Tripterygium wilfordii*, KM978213), *Pg*HMGS (*Panax ginseng*, ADI80347), *Hb*HMGS (*Hevea brasiliensis*, BAF98279), *Cr*HMGS (*Catharanthus roseus*, AEC13715), *Ca*HMGS (*Camptotheca acuminate*, ACD87446), *Cs*HMGS (*Camellia sinensis*, AFC34137), *At*HMGS (*Arabidopsis thaliana*, NP_192919), *Bj*HMGS (*Brassica juncea*, AAG32924), and *Sl*HMGS (*Solanum lycopersicum*, ABX55778).

The amino acid sequences were retrieved from the GenBank. ▲ The *Tw*HMGS sequence. The accession numbers are as follows: *Tripterygium wilfordii*, KM978213; *Panax ginseng*, ADI80347; *Hevea brasiliensis*, BAF98279; *Catharanthus roseus*, AEC13715; *Camptotheca acuminate*, ACD87446; *Camellia sinensis*, AFC34137; *Arabidopsis thaliana*, NP_192919; *Brassica juncea*, AAG32924; *Solanum lycopersicum*, ABX55778; *Ganoderma lucidum*, AFM91095; *Saccharomyces cerevisiae*, CAA65437; and *Oryza brachyantha*, ABG73437.

### 2.3. Expression of TwHMGS in the Suspension Cells

**Figure 5:**
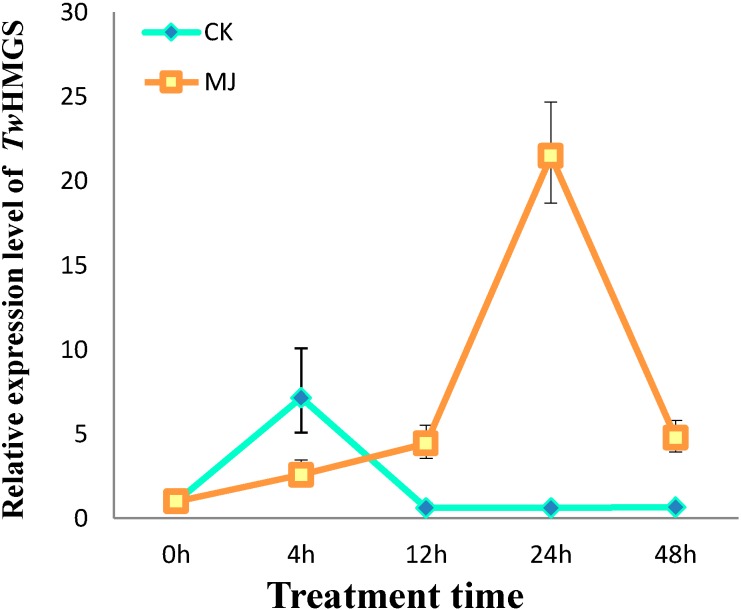
Expression levels of *TwHMGS* in suspension cells after MeJA treatment.

CK, the control group; MJ, the MJ treatment group.

Quantitative real-time PCR revealed that *TwHMGS* expression was induced by 50 μM MeJA in suspension cell cultures. The relative expression level of *TwHMGS* in the induced group reached the highest level at 24 h after the treatment (21.48-fold that in the control group). However, the expression of *TwHMGS* was not consistent in the control group; the expression level in the control group was significantly elevated 4 h after treatment relative to the other time points (Figure 5).

### 2.4. Expression of TwHMGS in Tissues

**Figure 6:**
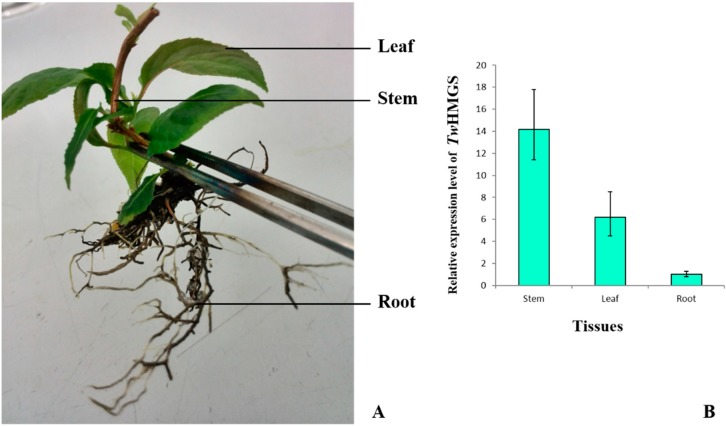
Expression of *TwHMGS* in tissues. (**A**) The *T. wilfordii* aseptic seedling and the tissue samples; (**B**) The relative expression of *TwHMGS* in different tissues.

*TwHMGS* was detected in all three kinds of the aseptic *T. wilfordii* seedling tissues. *TwHMGS* exhibited the highest expression level in the stem, followed by the leaf. The expression level in the root was the lowest (Figure 6).

### 2.5. Discussion

Research into the biosynthesis of terpenoids in medicinal plants supports our understanding of the corresponding biosynthetic pathways and provides the possibility of regulating the accumulation of target metabolites. In this study, we examined the terpenoid biosynthesis pathway in *Tripterygium wilfordii* by first cloning a *HMGS* gene. The *TwHMGS* was subsequently transformed into a proper knockout yeast strain to identify the gene function. Furthermore, the induced expression and tissue expression patterns were examined.

Because HMGS is involved in an important step in the MVA pathway, it has been cloned from many plants, including *Hevea brasiliensis* [22], *Taxus media* [23], and *Arabidopsis thaliana* [24]. *Tw*HMGS is highly similar to HMGS sequences from other organisms. Prior catalysis studies have demonstrated that three amino acid residues are absolutely conserved in HMGS: *Cys, His,* and *Asn* [25]. These residues are also conserved in *Tw*HMGS. The *TwHMGS* complement assays revealed that the expression of *TwHMGS* provides basic material for yeast survival. Gal in the YPG + G418 medium stimulated the expression of *TwHMGS*, whereas no expression was observed in the YPD + G418 medium due to the absence of Gal in YPD + G418 medium.

The expression pattern of *TwHMGS* in aseptic seedlings revealed that the gene is expressed in all tissues, but is expressed at higher levels in the stems. Interesting, though the woody stem of *Tripterygium wilfordii* can be used in traditional medicine, the root is the most commonly used tissue. This result suggest that further studies are needed to investigate the best tissue for medicinal use of the plant by chemical quantity analysis. HMGS expression in cell suspensions was induced at 12 h, 24 h and 48 h after MeJA treatment. The relative expression peaked at 24 h and fell by 48 h. However, the expression level of the CK group also exhibited a transient increase. This result may reflect expression level changes after treatment with DMSO solvent. With increasing treatment time, the expression level of the CK group was similar with that at 0 h.

In conclusion, the cloning of *TwHMGS* provides a foundation for further studies of the biosynthesis of terpenoids in *Tripterygium wilfordii*. The sequence provides a basic gene element for the generation of bioactive terpenoids in *Tripterygium wilfordii*. Researchers have already produced natural bioactive products in yeast or *E. coli* using synthentic biology strategies [26,27]. The correlation between *HMGS* expression and compound occurrence needs to be investigated to produce the bioactive terpenoids on a large scale. Meanwhile, *Tw*HMGS is also a committed enzyme in the biosynthetic pathway engineering of active terpenoids in *Tripterygium wilfordii*. Further research will be necessary to clarify the process of catalysis in plants to fully understand the biosynthesis of metabolites. And other relevant genes involved in terpenoids synthesis in *Tripterygium wilfordii* need to be detected.

## 3. Experimental Section

### 3.1. Plant Material

*T. wilfordii* aseptic seedling cultures were grown in Murashige and Skoog (MS) medium containing 30 g/L sucrose and 8 g/L agar with 0.2 mg·L^−1^ β-indoleacetic acid (IAA), 0.5 mg·L^−1^ kinetin (KT), and 1.5 mg·L^−1^ 6-benzylaminopurine (6-BA). The aseptic seedlings were cultured at 25 ± 1 °C with 16 h lighting/8 h dark. The *T. wilfordii* cell suspensions were cultured in MS medium containing 30 g/L sucrose and 8 g/L agar with 0.5 mg·L^−1^ 2,4-dichlorophenoxyacetic acid (2,4-D), 0.1 mg·L^−1^ KT, and 0.5 mg·L^−1^ indole-3-butytric acid (IBA). All suspension cell cultures were maintained at 25 ± 1 °C with shaking at 120 rpm in the dark.

### 3.2. RNA Isolation

The 10-day-old *T. wilfordii* suspension cells were treated with MeJA for 0, 4, 12, 24 or 48 h at a final concentration of 1 mM. Subsequently, the suspension cells were harvested for RNA isolation. The root, stem and leaf of the aseptic seedling of *T. wilfordii* were separately harvested for RNA isolation as well. The total RNA was isolated using the cetyltrimethylammonium bromide (CTAB) method [28].

### 3.3. Strains

The HMGS knockout yeast strain YSC6274 (BY4743; *MAT a/α*; *his3D1/his3D1*; *leu2D0/ leu2D0*; *lys2D0/LYS2*; *MET15/met15D0*; *ura3D0/ura3D0*; *yml126c: kanMX4/YML126c*) was obtained from the GE Healthcare Yeast Knock Out Strain Collection (GE Healthcare Dharmacon, Inc., Lafayette, CO, USA).

### 3.4. Cloning of TwHMGS Full-Length cDNA

Total RNA was reverse transcribed into first-stand cDNA with SMARTer™ RACE cDNA Amplification Kit (Clontech Laboratories Inc., Mountain View, CA, USA) according to the manufacturer’s instructions. The full-length primers (HMGS-F: 5'-GATGGACCTTTCTTGTTTCCTCTA-3' and HMGS-R: 5'-GAGAAGCGAAAAATACACTGAAAAC-3') were designed based on the transcriptome sequencing data of *Tripterygium wilfordii* obtained previously. The product was purified and cloned into the pMD19-T vector (Takara Biotechnology (Dalian) Co., Ltd., Dalian, China). The vector was transformed into *E. coli* DH5α cells and cultured in Luria-Bertani medium at 37 °C in dark. The positive colonies were sequenced and assembled to verify the correct *TwHMGS* insertion.

### 3.5. Bioinformatics Analysis

The nucleotide sequence was BLAST on NCBI (National Center for Biotechnology Information). The open reading frame (ORF) and amino acid sequence of *Tw*HMGS were deduced using the ORF finder [29]. *Tw*HMGS and other HMGS sequences downloaded from GenBank were aligned, and the phylogenetic tree was constructed by the neighbour-joining method using MEGA5.1. The 3-dimensional structural modelling was predicted by Swiss-Model.

### 3.6. Construction of the pYES2-TwHMGS Expression Vector

The fragment containing *TwHMGS* ORF and the proper restriction enzyme sites was amplified using HMGS-BamHI (5'-cgcggatccATGGCAAAGAATGTCGGG-3') and HMGS-EcoRI (5'-ccggaattc AGTGACCATTGGCAACTGC-3') as primers. The product and the empty pYES2 vector were digested, gel-purified and ligated. Positive colonies were confirmed by PCR and sequencing. The constructed *pYES2*-*Tw*HMGS plasmids were extracted and stored at −20 °C before transformation into yeast.

### 3.7. Complementation Assays

The glycerol stock of YSC6274 was activated twice on YPD medium (1% yeast extract, 2% Bacto-Peptone and 2% glucose) at 30 °C in advance. *pYES2-TwHMGS* was transformed into YSC6274 using the Frozen-EZ Yeast Transformation II Kit (ZymoResearch, Irvine, CA, USA). Meanwhile the empty pYES2 plasmid was also transformed as a control. The transformants were spotted on SC (-Ura) medium (6.7% yeast nitrogen base w/o amino acid, 2% galactose), and the positive colonies was further verified by PCR. Subsequently, the transformed diploid cells were forced to sporulate and form the haploid cells containing *pYES2-TwHMGS*. The haploid cells were selected on YPG + G418 medium (1% yeast extract, 2% Bacto-Peptone, 2% galactose and 200 ml/L geneticin). The diploid and the haploid cells were grown on YPD + G418 (1% yeast extract, 2% Bacto-Peptone, 2% glucose and 200 ml/L geneticin) and YPG + G418 medium separately to compare the grown states. SC (-Ura) + 5-FOA medium (6.7% yeast nitrogen base w/o amino acid, 2% galactose and 0.1% 5-fluoroorotic acid) was used to analyse plasmid loss.

### 3.8. Quantitative Real-Time PCR

Total RNA was used to synthesise the first strand cDNA with TIANScript II RT Kit (Tiangen Biotech (Beijing) Co., Ltd., Beijing, China) according to the manufacturer’s protocols. The relative mRNA levels were estimated with the Applied Biosystems 7300 Real Time PCR System (Applied Biosystems, Grand Island, NY, USA) using KAPA SYBR^®^ FAST qPCR Kit (KAPA Biosystems, Wilmington, MA, USA). The expression of the target gene was normalised according to the expression of the housekeeping gene, β-actin, and evaluated using the 2^−ΔΔCt^ method [30]. There were three samples in each group and each sample was repeated for three times to insure the credibility of the data. The real-time PCR primers were designed by Primer Premier 5.0 as follows: β-actin F (5'-AGGAA CCACCGATCCAGACA-3') and β-actin R (5'-GGTGCCCTGAGGTCCTGTT-3'), and HMGS-qF (5'-CTGGAGGTAGGGAGCGAGAC-3') and HMGS-qR (5'-CCATAGCAGGCATTGGTTGA-3').

## 4. Conclusions

In this paper, the *HMGS* gene in *Tripterygium wilfordii* was cloned and characterised using yeast complement assays. The *TwHMGS* presents high similarity with other *HMGS*s and belong to the HMGS family. The tissues expression of HMGS in *Tripterygium wilfordii* aseptic seedlings and the expression in *Tripterygium wilfordii* suspension cells induced by Methyl jasmonate (MeJA) were detected.
